# Ablation Index Predicts Successful Ablation of Focal Atrial Tachycardia: Results of a Multicenter Study

**DOI:** 10.3390/jcm11071802

**Published:** 2022-03-24

**Authors:** Paolo Compagnucci, Antonio Dello Russo, Marco Bergonti, Matteo Anselmino, Giulio Zucchelli, Alessio Gasperetti, Laura Cipolletta, Giovanni Volpato, Ciro Ascione, Federico Ferraris, Yari Valeri, Maria Grazia Bongiorni, Andrea Natale, Claudio Tondo, Gaetano Maria De Ferrari, Michela Casella

**Affiliations:** 1 Cardiology and Arrhythmology Clinic, University Hospital “Ospedali Riuniti”, 60126 Ancona, Italy; antonio.dellorusso@gmail.com (A.D.R.); cipollettalaura@gmail.com (L.C.); giovol@live.it (G.V.); yarivaleri1@gmail.com (Y.V.); michelacasella@hotmail.com (M.C.); 2 Department of Biomedical Sciences and Public Health, Marche Polytechnic University, 60126 Ancona, Italy; alessio.gasperetti93@gmail.com; 3 Operation Unit of Arrhythmology, Centro Cardiologico Monzino, Istituto di Ricovero e Cura a Carattere Scientifico (IRCCS), 20138 Milan, Italy; bergmar21@gmail.com (M.B.); ciroascione92@gmail.com (C.A.); claudio.tondo@cardiologicomonzino.it (C.T.); 4 Department of Clinical Sciences and Community Health, University of Milan, 20138 Milan, Italy; 5 Division of Cardiology, “Città della Salute e della Scienza di Torino” Hospital, Department of Medical Sciences, University of Turin, 10126 Turin, Italy; anselmino.matteo@unito.it (M.A.); federico_ferraris@yahoo.it (F.F.); gaetanomaria.deferrari@unito.it (G.M.D.F.); 6 Second Division of Cardiovascular Diseases, Cardio-Thoracic and Vascular Department, University Hospital of Pisa, 56124 Pisa, Italy; g.zucchelli@ao-pisa.toscana.it (G.Z.); m.g.bongiorni@med.unipi.it (M.G.B.); 7 Texas Cardiac Arrhythmia Institute, St. David’s Medical Center, Austin, TX 78705, USA; dr.natale@gmail.com; 8 Department of Clinical, Special and Dental Sciences, Marche Polytechnic University, 60126 Ancona, Italy

**Keywords:** focal atrial tachycardia, catheter ablation, electroanatomical mapping, ablation index

## Abstract

A radiofrequency energy lesion transmurality marker incorporating power, contact force, and time (Ablation Index, AI) was shown to be associated with outcomes of catheter ablation (CA) of multiple arrhythmias, but was never systematically assessed in the CA of focal atrial tachycardias (AT). We aimed to evaluate the role of AI as a predictor of outcomes in focal AT CA, and therefore, retrospectively included 45 consecutive patients undergoing CA for focal AT in four referral electrophysiology laboratories. Clinical and procedural information were collected. For each patient, maximum and mean (by averaging maximum AI values for each radiofrequency ablation lesion) AI were measured. The primary outcome was focal AT-free survival, and was systematically assessed with periodical Holter monitors or cardiac implantable electronic devices. CA was acutely effective in each case; however, 20% (*n* = 9) of the study population experienced a focal AT recurrence over a median follow-up of 288 days. Both maximum and mean AI values were significantly higher among patients without AT recurrences (maximum AI = 568 ± 91, mean AI = 426 ± 105) than in patients with AT relapses (maximum AI = 447 ± 142, mean AI = 352 ± 76, *p* = 0.036, and *p* = 0.028, respectively). The optimal cutoffs associated with freedom from recurrences were 461 for maximum AI (sensitivity, 0.89; specificity, 0.56) and 301 for mean AI (sensitivity, 0.97; specificity, 0.44). In a time-to-event analysis, maximum AI was significantly associated with survival free from AT recurrence (*p* = 0.001), whereas mean AI was not (*p* = 0.08). In summary, maximum AI is the best procedural parameter associated with the outcomes of CA for focal AT, and may help standardize the procedural approach.

## 1. Introduction

Focal atrial tachycardia (AT) is an uncommon type of supraventricular tachycardia, resulting from enhanced automaticity, triggered activity, or localized micro-reentry [1]. Although patients with AT may be completely asymptomatic, AT may result in palpitations, poor quality of life, or induce a form of heart failure known as tachycardiomyopathy [1]. Catheter ablation (CA) with radiofrequency energy has evolved to become the first-line guideline-recommended therapy in recurrent and/or incessant cases [1].

Recently, an ablation lesion quality marker that incorporates radiofrequency power (Watts), contact force (grams), and time (seconds), known as Ablation Index (AI) was introduced [2]. AI reflects lesion transmurality, and not unexpectedly was found to correlate with CA outcomes in atrial fibrillation [3,4,5], atrial flutter [6], and premature ventricular complexes [7,8].

As of today, no study has examined whether AI is also associated with outcomes of CA in patients with focal AT, and the optimal AI targets outside pulmonary veins and cavotricuspid isthmus are yet to be determined. We conducted this analysis to test the hypothesis that AI can be used as a predictor of CA success in focal AT, and to suggest optimal target values.

## 2. Materials and Methods

### 2.1. Study Population

This was a multicenter, retrospective observational study of patients undergoing radiofrequency energy CA for focal AT in four tertiary-level electrophysiology Italian centers, using the CARTO3 electroanatomical mapping system (Biosense Webster, Diamond Bar, CA, USA) and a contact-force sensing catheter (Thermocool SmartTouch or Thermocool SmartTouch SF, Biosense Webster, Diamond Bar, CA, USA) equipped with an AI module. We included consecutive patients undergoing a first-time procedure, as well as patients with previous catheter ablation for supraventricular arrhythmias, but only if the index focal AT originated from a site not previously ablated. Each procedure performed between 2017 and 2021 was retrospectively reviewed, and a minimum follow-up of six months was required for inclusion in the analysis. The research reported in this paper adhered to institutional standards, national legal requirements, and the Helsinki Declaration as revised in 2013. Every patient provided his/her written informed consent.

### 2.2. Electrophysiology Study and Catheter Ablation

The presumed site of origin of the AT was preliminarily determined according to *p*-wave morphology, following a previously published algorithm [9]. Among patients receiving anticoagulants, CA was performed on uninterrupted anticoagulation in case of vitamin K antagonist, or after skipping the morning dose in case of direct oral anticoagulants. Antiarrhythmic drugs were discontinued at least five half-lives prior to the procedure, with the exception of amiodarone. All procedures were performed in the fasted awake state, with minimal use of sedation, and from the right femoral venous access. In case of a presumed left atrial origin, the left atrium was accessed with a single transseptal puncture, and an activated clotting time of >300 s was maintained throughout the procedure.

In case of absence of spontaneous AT the beginning of the procedure, burst and/or programmed electrical stimulation were performed, and repeated during isoproterenol infusion, until AT was induced. First, a macroreentrant mechanism was excluded according to classic electrocardiograpy and electrophysiology criteria [10]. After confirming the diagnosis of focal AT, a three-dimensional reconstruction of the chamber of interest (right or left atrium) was obtained with the CARTO3 electroanatomical mapping system (Biosense Webstern, Diamond Bar, CA, USA), using a contact force sensing catheter (Thermocool SmartTouch or Thermocool SmartTouch SF, Biosense Webster, Diamond Bar, CA, USA), and a multielectrode catheter as needed. Color-coded local activation time (LAT) maps of the atrium were reconstructed, by annotating LAT relative to a catheter positioned into the coronary sinus. The origin of the focal AT was defined as the site showing the earliest bipolar electrogram, with a LAT of >20–30 milliseconds prior to the onset of the *p*-wave. Furthermore, using unipolar recordings, the site of origin was confirmed for having a pure negative (QS morphology) deflection.

Radiofrequency energy was delivered with the contact-force sensing open-irrigated ablation catheter in the area of interest, by choosing radiofrequency power (25–40 W), contact force (≥3 gr), and ablation time (30–120 s) according to the operator’s preference and experience and to the response of the focal AT. Irrigation flow rate was set at 2 mL/min during mapping, and at 17–30 mL/min (Thermocool SmartTouch, Biosense Webster, Diamond Bar, CA, USA) or 8–15 mL/min (Thermocool SmartTouch SF, Biosense Webster, Diamond Bar, CA, USA) during radiofrequency delivery, following the recommendations of the manufacturer.

### 2.3. Ablation Index

**Figure 1:**
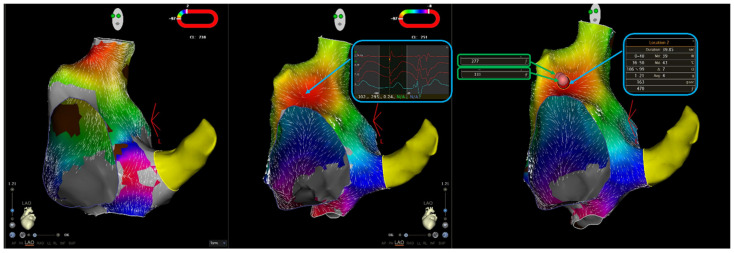
Ablation of a focal atrial tachycardia originating from the right atrial anteroseptal area. The left panel shows right atrial activation mapping in a sinus rhythm; the red zone indicates the sinus node. The central panel shows activation mapping during atrial tachycardia, with earliest local activation time in the interatrial septum, where a QS unipolar electrogram is recorded. The right panel shows ablation lesions; two radiofrequency applications were delivered, with the creation of three Visitags. The maximum ablation index (AI) for lesion 1 (blue box) is 470, whereas the maximum AI for lesion 2 (green boxes) is 331. Therefore, in the retrospective analysis of the case, maximum AI was 470, mean ablation index was 401 (calculated the average of the two maximum AI values for lesions 1 and 2), and minimum ablation index was 331.

Every ablation lesion delivered in the site of origin of the focal AT was examined, and the location of the lesion was automatically annotated using a dedicated module on the CARTO electroanatomical mapping system (Visitag, Biosense Webster, Diamond Bar, CA, USA) with the following settings: catheter stable for at least 3 s within a range of 3 mm; minimum contact force of 3 g for at least 25% of the stability time. The maximum AI value was determined for each case (Figure 1). Furthermore, mean (by averaging AI values for each radiofrequency application in a given patient) and minimum (by taking the radiofrequency energy delivery with the lowest maximum AI value in a given patient) AI values were measured (Figure 1). Operators reviewing CARTO files were blinded to the patients’ outcomes.

### 2.4. Outcomes

The primary outcome of the study was freedom from recurrent focal AT lasting 30 s or more. Secondary outcomes were an occurrence of other supraventricular arrhythmias, including atrial flutter and atrial fibrillation, as well as procedural complications. Arrhythmic outcomes were assessed with clinical follow-ups, electrocardiography, and 24 h Holter monitor recordings three months after the procedure and yearly thereafter in asymptomatic patients, or earlier in the case of symptoms, without any blanking period. In addition, arrhythmia recurrences were continuously assessed by cardiac implantable electronic devices, when available.

### 2.5. Statistical Analysis

The Shapiro–Wilk’s test was used to check continuous variables for normality; non-normal variables were expressed as median (1st–3rd quartile), whereas categorical variables were reported as counts and percentages. The primary outcome was assessed in a time-to-event fashion, with the Kaplan–Meier method, and the role of AI as a predictor of focal AT recurrence was tested with univariable Cox proportional hazard regression, and results were summarized by reporting hazard ratios (HR) and 95% confidence intervals. Furthermore, differences in AI values between patients experiencing a primary outcome event and patients not experiencing a primary outcome event were analyzed with the Student’s *t* test or the Wilcoxon rank-sum test for normally and non-normally distributed variables, respectively. Discrimination ability of AI was measured with area under the receiver operating characteristic curve, and the optimal AI cutoff value was identified as that maximizing Youden’s index (i.e., sensitivity + specificity−1). An alpha level <0.05 was considered statistically significant, and the software RStudio (RStudio Inc., Boston, MA, USA) was used for statistical analysis.

## 3. Results

### 3.1. Patients

Table 1 shows the characteristics of the patients at baseline. We included 45 consecutive patients. The mean age of the cohort was 49 ± 17 years, and 60% were female. Eleven patients (24%) had a history of structural heart disease, mainly valvular heart disease (*n* = 3) or congenital heart disease (*n* = 4) with prior surgical procedures. The median left ventricular ejection fraction was 60 (58–65), the median indexed left atrial volume was 32 mL/m^2^ (24–50 mL/m^2^), and the mean right atrial area was 20 ± 7 cm^2^. Besides focal AT, 13 patients (29%) had a history of paroxysmal/persistent atrial fibrillation, ten (22%) had a history of cavotricuspid isthmus-dependent atrial flutter, and 13 (29%) had frequent premature atrial complexes (PACs) at baseline Holter monitor recordings, with a mean 24 h burden of 11471 ± 7412 PACs. Fourteen patients (33%) previously underwent CA with pulmonary vein isolation (*n* = 6, 13%), ablation of focal AT originating from a different site (*n* = 6, 13%), cavotricuspid isthmus line (*n* = 3, 7%), or slow pathway ablation (*n* = 1, 2%).

Prior to CA, 22 subjects (49%) were receiving class Ic (*n* = 21) and/or class III (*n* = 3) antiarrhythmic medications. Continuous rhythm monitoring was available in 13 patients (29%), who had dual-chamber pacemakers (*n* = 7, 16%) or loop recorders (*n* = 6, 13%).

### 3.2. Electrophysiological and Procedural Findings

**Figure 2:**
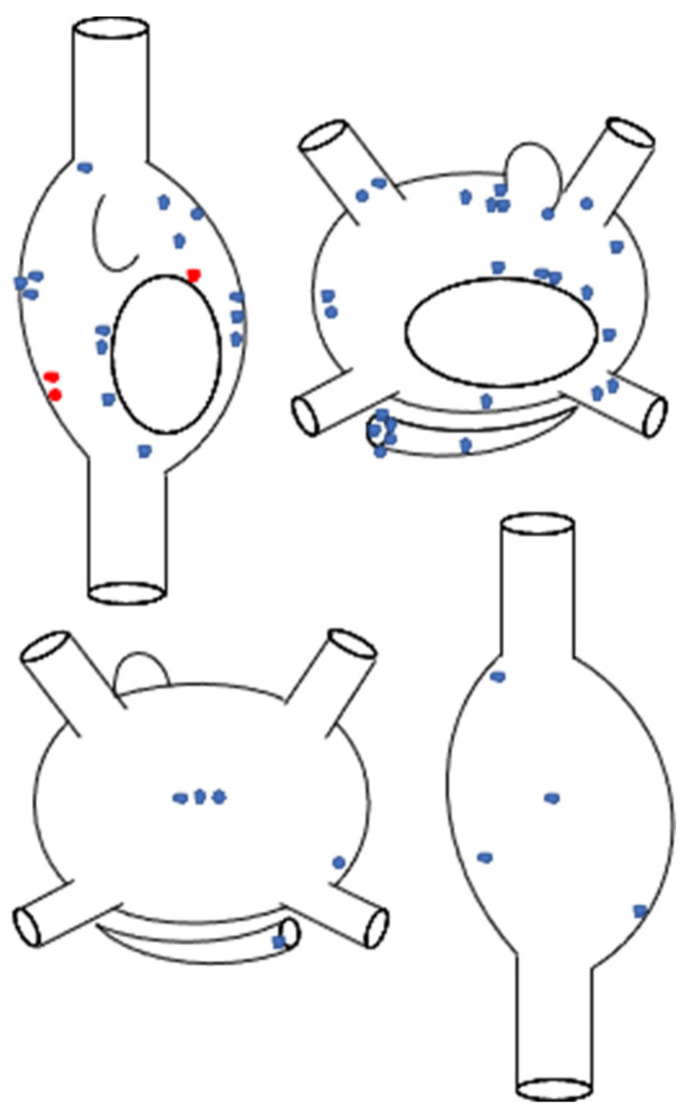
Schematic representation of the anatomic distribution of focal atrial tachycardias (*n* = 51), according to the sites of successful ablation. Superior panel: anteroposterior biatrial view; inferior panel: posteroanterior biatrial view. Blue stars represent automatic atrial tachycardias (*n* = 48), whereas red stars represent micro-reentrant atrial tachycardias (*n* = 3).

Fifty-one different focal ATs were identified and mapped, with one focal AT mapped and ablated in 41 patients (91%), two ATs in three patients (7%), and four ATs in one patient (2%). Regarding the anatomic location, 27 focal ATs (53%) in 26 patients (58%) originated from the right atrium, whereas 24 ATs (47%) were mapped and ablated in the left atrium in 19 patients (42%). The specific site(s) of origin of focal ATs in our cohort are represented in Figure 2. The mechanism of focal ATs was enhanced automaticity/triggered activity in 48 cases (94%), and micro-reentry in three (6%).

The median procedural time measured 120 min (93–150 min), and the median fluoroscopy time was 11 min (5–20 min). A median of eight (3–14) radiofrequency energy applications was delivered per patient, with a median of 11 (4–21) Visitag points and a median radiofrequency time of 4 min (2–8 min).

### 3.3. Outcomes

**Figure 3:**
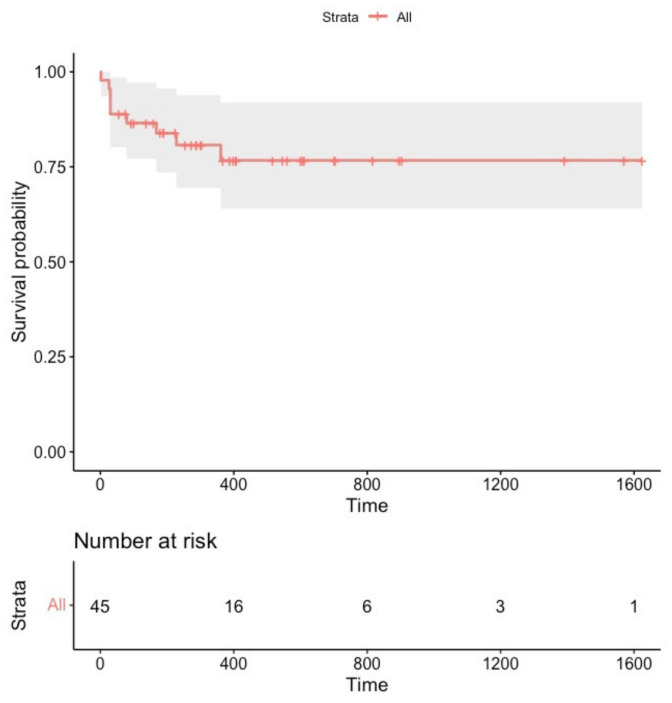
Survival free from sustained focal atrial tachycardia recurrence in the overall study population (*n* = 45).

Focal ATs were terminated during the procedure in each patient. During a median follow-up of 288 days (160–560), nine patients (20%) experienced focal AT recurrences (Figure 3). The clinical characteristics of patients experiencing primary outcome events are presented in the Supplementary Table S1.

There were no complications during the electrophysiology procedure; however, during hospitalization, one patient who underwent CA of the right atrial lateral wall and of the coronary sinus ostium developed symptomatic sinus node disease and was implanted with a pacemaker, whereas another subject with CA at the left pulmonary vein carina developed asymptomatic sinus arrests, for which he received an implantable loop recorder.

At the last follow-up, 30 patients (71%) were not on antiarrhythmic medications, whereas 13 subjects (29%) were still receiving class Ic (*n* = 12) or class III (*n* = 1) drugs.

### 3.4. Ablation Index and Outcomes

In our sample, the mean value of maximum AI measured 544 ± 112, the mean value of mean AI was 411 ± 103, and the median value of minimum AI measured 244 (194–338).

**Figure 4:**
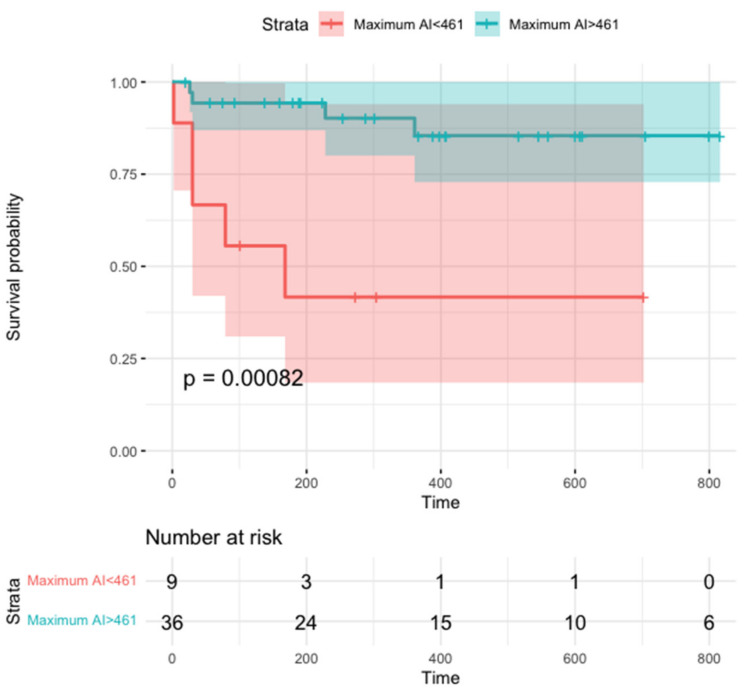
Survival free from focal atrial tachycardia recurrence according to maximum ablation index. The 461 value was chosen for being the optimal cutoff associated with freedom from recurrent AT according to Youden’s index (see text).

In a time-to-event analysis, maximum AI was significantly related to the primary outcome (HR, 0.99 (95% CI, 0.98–0.99); *p* = 0.001, Figure 4), whereas a similar association was not observed for mean or minimum AI (HR, 0.99 (95% CI, 0.98–1.00), *p* = 0.08 and HR, 1.00 (95% CI, 0.99–1.00), *p* = 0.50, respectively).

Furthermore, maximum AI and mean AI values were significantly higher in patients not experiencing focal AT recurrences, as compared to patients with recurrences (568 vs. 447, *p* = 0.036 and 426 vs. 352, *p* = 0.028, respectively); no significant difference was observed for minimum AI (*p* = 0.60). Other procedural parameters, including procedural/fluoroscopy time, the number or radiofrequency applications, the number of Visitags, and total radiofrequency time, were similar between patients experiencing and those not experiencing AT recurrences (Table 2).

**Figure 5:**
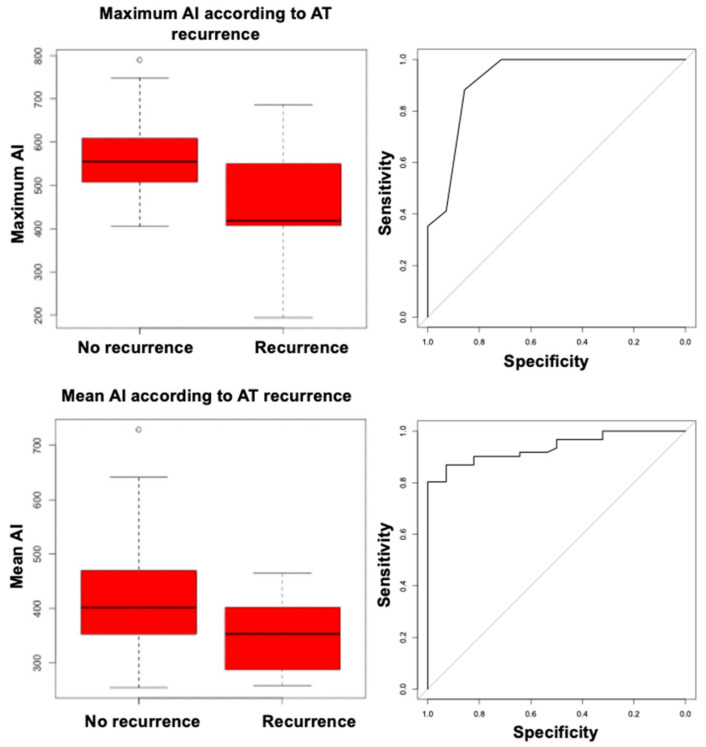
Box-plots of maximum ablation index (**upper left**) and mean ablation index (**lower left**) according to atrial tachycardia recurrence; receiver operating characteristic curves for maximum ablation index (**upper right**) and mean ablation index (**lower right**) as predictors of atrial tachycardia recurrence.

In order to retrospectively identify the optimal AI cutoff associated with AT recurrences, we performed receiver operating characteristic (ROC) curve analysis, and found that both maximum AI and mean AI had good discrimination ability for recurrent AT, with areas under the ROC curve (AUC) of 0.92 (95% CI, 0.85–1.00) and 0.94 (95% CI, 0.89–0.98), respectively (Figure 5); discrimination ability was statistically similar (DeLong test’s *p* = 0.74).

The optimal cutoffs associated with freedom from recurrent AT measured 461 for maximum AI (sensitivity, 0.89; specificity, 0.56) and 301 for mean AI (sensitivity, 0.97; specificity, 0.44). When separately analyzing patients who underwent right atrial CA (*n* = 26), maximum AI was still associated with the primary outcome (HR, 0.99 (95% CI, 0.98–0.99), Bonferroni corrected *p* = 0.02), with an optimal cutoff of 480 (sensitivity, 0.90; specificity, 0.83); patients without focal AT recurrences had significantly higher maximum AI values than patients experiencing recurrences (594 vs. 395, Bonferroni corrected *p* = 0.02). A trend for a similar association was found for mean AI (HR, 0.98 (95% CI, 0.97–0.99), Bonferroni corrected *p* = 0.08), and mean AI values were insignificantly lower in subjects experiencing a primary outcome event (287 vs. 360, Bonferroni corrected *p* = 0.057). The association of maximum and mean AI with the primary outcome could not be proven in the limited subset of patients who underwent left atrial CA (*n* = 19, HR, 1 (95% CI, 0.99–1.02), *p* = 0.90 for maximum AI; HR, 1 (95% CI, 0.98–1.02), *p* = 0.92 for mean AI), in whom high AI values were reached on average (maximum AI, 539 ± 77; mean AI, 444 ± 75).

## 4. Discussion

### 4.1. Main Findings

To the best of our knowledge, this is the first study demonstrating that AI, a numerical proxy of lesion transmurality, is significantly linked to outcomes of CA for focal AT. Although AI was previously found to be predictive of long-lasting pulmonary vein isolation among patients undergoing CA for atrial fibrillation, and to be associated with CA efficacy in patients with idiopathic premature ventricular complexes [7,8], our retrospective data suggest for the first time that higher AI values may be predictive of AT recurrence-free survival.

Notwithstanding the tremendous improvements in knowledge and techniques in the fields of electroanatomical mapping and CA that we witnessed in recent years [11], CA for focal AT still has suboptimal outcomes, even in experienced hands, with most series reporting a recurrence rate of 10–20% [1,12].

Rarely, focal ATs cannot be mapped from the endocardium or coronary sinus, and epicardial arrhythmogenic structures (such as Bachmann’s bundle) are implied in AT pathogenesis [13]. Even when ATs are successfully mapped and terminated/suppressed with radiofrequency energy CA, it is not uncommon to see later recurrences, when the automatic focus/microreentrant circuit recovers from the transient radiofrequency energy-induced stunning. In this respect, when focal AT originate deep in the atrial wall, more energy (power and time) applied more efficiently (adequate catheter-tissue contact) may be required to obtain procedural success; in other words, deeper ablation lesions may be needed to achieve long-term AT suppression, and at least in some patients, recurrence of AT may be secondary to inadequate lesion transmurality.

Our data show that maximum AI is associated with the effectiveness of CA for AT in both time-to-event and regression analyses, as compared to mean AI, which is only significantly linked to CA efficacy in regression analysis. Although the limited number of patients enrolled may have played a role in this finding, with focal AT being an arrhythmia due to a localized automatic focus/micro-reentrant circuit, maximum AI may be a stronger predictor of outcomes than mean AI: a single transmural radiofrequency energy lesion right on the AT’s site of origin may be more effective than multiple non-transmural or imprecise radiofrequency energy applications. Furthermore, when stacking multiple radiofrequency energy pulses on the same spot, the relationship between AI and lesion transmurality has not been well explored and may not be well captured by simply summing AI values from each radiofrequency energy delivery. These results closely mimic what we recently found for CA of premature ventricular complexes, another focal arrhythmia [7,8].

Our results show that AI values similar to those previously found to be associated with long-lasting pulmonary vein isolation are also associated with better outcomes in AT CA, with most AT foci located outside pulmonary veins (Figure 1). This finding is not surprising, given that most autopsy and imaging studies investigating atrial wall thickness were unable to show clear regional variations between pulmonary vein antra, which are the target of CA for atrial fibrillation, and other atrial segments, while also showing a certain degree of inter-patient variability [14]. It is to be noted that in our sample, no case of pericardial effusion or intraprocedural mechanical complication was reported, thus supporting the safety of the ablation approach, which could be theoretically reproduced by achieving similar AI values.

When separately analyzing patients undergoing left atrial CA, we were unable to demonstrate an association between AI and procedural outcomes. However, this analysis was clearly underpowered (only 19 patients underwent left atrial CA in our study), and high AI values were obtained on average in this subset, supporting the possibility that focal AT recurrences were more related to the progression of left atrial substrate than to lack of lesion transmurality.

AI may serve as a reproducible, operator-independent quantitative reference at the time of AT ablation, and the availability of such a lesion quality marker could represent one additional reason to consider using electroanatomical mapping systems for supraventricular arrhythmia ablation, apart from the well-known reduced exposure to ionizing radiations [15], and the improved long-term outcomes for some types of supraventricular arrhythmias [16]. In case our results are confirmed in larger, prospective studies, AI may become a critical element in the process of standardization of CA of focal AT, thus limiting the impact of inter-operator variability in long-term procedural success.

### 4.2. Study Limitations

Our study has several limitations. First, due to the limited number of patients we included, we were unable to derive segment-specific cutoffs for AI values. However, as previously discussed, most studies on atrial wall thickness did not show significant regional variations [14], with only some authors reporting left atrial roof segments as the thinnest [17], thus supporting the concept that a similar lesion transmurality may be required for most AT sites of origin. Second, our study had a retrospective design, and further prospective validation is clearly needed before AI cutoffs may be clinically applied. Third, during follow-up, almost half of our study population was still on antiarrhythmic drugs, presumably due to the common coexistence of focal AT with other arrhythmias, especially atrial fibrillation. This finding may have influenced our results.

## 5. Conclusions

We provided the first systematic assessment of AI in focal AT ablation, and found that maximum AI may serve as a good predictor of long-term CA success. In the case that our results are confirmed in larger, prospective studies, AI may become a procedural standard for focal AT CA, and help curtail operator dependency.

## Figures and Tables

**Table 1:** Baseline clinical characteristics. Categorical variables are presented as *n* (%).

	Overall (*n* = 45)
Age—years (SD)	49 (17)
Male sex—no. (%)	27 (60)
CHA_2_DS_2_-VASc score—median (1st–3rd quartile)	1 (1–2)
HASBLED score—median (1st–3rd quartile)	0 (0–1)
EHRA score at baseline—median (1st–3rd quartile)	1 (0–2)
Structural heart disease—no. (%)	11 (24)
Congenital heart disease s/p surgical procedure—no. (%)	4 (9)
Valvular heart disease s/p surgical procedure—no. (%)	2 (4)
Mitral valve disease s/p mitral valve repair plus coronary artery disease—no. (%)	1 (2)
Myocarditis—no. (%)	1 (2)
Dilated cardiomyopathy—no. (%)	1 (2)
Restrictive cardiomyopathy plus coronary artery disease—no. (%)	1 (2)
Hypertensive heart disease—no. (%)	1 (2)
Echocardiography: Left ventricular ejection fraction—% (1st–3rd quartile) Indexed left atrial volume—ml/m^2^ (1st–3rd quartile) Right atrial area—cm^2^ (SD)	60 (58–65) 32 (24–50) 20 (7)
History of atrial fibrillation—no. (%)	13 (29)
History of atrial flutter—no. (%)	10 (22)
Frequent PACs—no. (%)	13 (29)
Frequent PACs burden—no./24 h (SD)	11471 (7412)
Prior catheter ablation:	13 (29)
Prior pulmonary vein isolation—no. (%)	6 (13)
Prior focal AT ablation—no. (%)	3 (7)
Prior cavotricuspid isthmus ablation—no. (%)	3 (7)
Prior AVNRT ablation—no. (%)	1 (2)
Antiarrhythmic drugs at baseline:	
None	14 (31)
Class I	21 (47)
Class III	3 (7)
Class II	16 (36)
Class IV	3 (7)

Abbreviations: AT, atrial tachycardia; AVNRT, atrioventricular nodal reentrant tachycardia; EHRA, European heart rhythm association; PAC, premature atrial complex.

**Table 2:** Procedural parameters according to outcomes in the overall population.

	No Recurrence Group (*n* = 36)	Recurrence Group (*n* = 9)	*p*
Maximum AI—mean (SD)	568 (91)	447(142)	0.036
Mean AI—mean (SD)	426 (105)	352 (76)	0.0284
Minimum AI—median (1st–3rd quartile)	243 (196–338)	244 (161–320)	0.60
Procedural duration—min (1st–3rd quartile)	120 (90–150)	130 (120–150)	0.45
RF time—min (1st–3rd quartile)	5 (2–8)	3 (2–4)	0.50
Number of RF pulses—median (1st–3rd quartile)	10 (3–18)	6 (6–7)	0.30
VISITAG no.—median (1st–3rd quartile)	12 (4–21)	8 (7–17)	0.80
Fluoroscopy time—min (1st–3rd quartile)	13 (6–21)	10 (4–11)	0.38

Abbreviations: AI, ablation index; RF, radiofrequency.

## Data Availability

The data presented in this study are available in the article.

## Supplementary Materials

The following supporting information can be downloaded at: https://www.mdpi.com/article/10.3390/jcm11071802/s1, Table S1: Clinical characteristics of patients experiencing a primary outcome event.
