# Supplementary material for: Ablation Index Predicts Successful Ablation of Focal Atrial Tachycardia: Results of a Multicenter Study
Source: J Clin Med. 2022 Mar 24;11(7):1802. doi: 10.3390/jcm11071802 (PMC8999753; doi:10.3390/jcm11071802)
Supplement: Supplementary file 1 [file jcm-11-01802-s001.zip › jcm-1616488-supplementary.pdf]

Supplementary material

Supplementary Table S1. Clinical characteristics of patients experiencing a primary outcome event.

Patient no.	Age	Gender	BMI	CHA_2DS_2-VASc score	HASBLED score	Structural heart disease	History of other arrhythmias	Focal AT origin
**1**	72	F	22	3	2	None	Frequent PACs	Low-septal right atrium
**2**	72	M	31	2	2	None	AFib and frequent PACs	Posterior right atrium
**3**	46	F	22	1	0	None	None	High right atrium
**4**	49	F	24	3	1	None	AFib	Left atrial appendage base
**5**	49	F	20	1	0	Myocarditis	None	Coronary sinus os
**6**	78	M	26	2	1	None	Atrial flutter	Left interatrial septum
**7**	60	F	23	2	0	None	Atrial flutter	Posterior-inferior right atrium
**8**	55	M	22	1	1	Congenital Heart Disease	AFib, atrial flutter and frequent PACs	Right superior pulmonary vein
**9**	44	F	35	1	0	None	None	ParaHisian

Abbreviations: AFib, atrial fibrillation; BMI, body mass index; PACs, premature atrial complexes.
